# Mouse IDGenes: a reference database for genetic interactions in the developing mouse brain

**DOI:** 10.1093/database/bau083

**Published:** 2014-08-20

**Authors:** Michaela Matthes, Martin Preusse, Jingzhong Zhang, Julia Schechter, Daniela Mayer, Bernd Lentes, Fabian Theis, Nilima Prakash, Wolfgang Wurst, Dietrich Trümbach

**Affiliations:** ^1^Institute of Developmental Genetics, Helmholtz Zentrum München, German Research Center for Environmental Health, Ingolstädter Landstr. 1, 85764 Neuherberg, Germany, ^2^Technische Universität München-Weihenstephan, Lehrstuhl für Genetik, Emil-Ramannstr. 8, 85354 Freising, Germany, ^3^Institute of Diabetes and Regeneration Research, Helmholtz Zentrum München, German Research Center for Environmental Health, Ingolstädter Landstr. 1, 85764 Neuherberg, Germany, ^4^Institute of Computational Biology, Helmholtz Zentrum München, German Research Center for Environmental Health, Ingolstädter Landstr. 1, 85764 Neuherberg, Germany, ^5^Technische Universität München, Zentrum Mathematik, Boltzmannstr. 3, 85747 Garching, Germany, ^6^Max-Planck-Institute of Psychiatry, Kraepelinstr. 2-10, 80804 München, Germany, ^7^Deutsches Zentrum für Neurodegenerative Erkrankungen e. V. (DZNE), Standort München, Schillerstr. 44, 80336 München, Germany, ^8^Technische Universität München-Weihenstephan, Lehrstuhl für Entwicklungsgenetik, c/o Helmholtz Zentrum München, Ingolstädter Landstr. 1, 85764 Neuherberg, Germany and ^9^Munich Cluster for Systems Neurology (SyNergy), Adolf-Butenandt-Institut, Ludwig-Maximilians-Universität München, Schillerstr. 44, 80336 München, Germany

## Abstract

The study of developmental processes in the mouse and other vertebrates includes the understanding of patterning along the anterior–posterior, dorsal–ventral and medial– lateral axis. Specifically, neural development is also of great clinical relevance because several human neuropsychiatric disorders such as schizophrenia, autism disorders or drug addiction and also brain malformations are thought to have neurodevelopmental origins, i.e. pathogenesis initiates during childhood and adolescence. Impacts during early neurodevelopment might also predispose to late-onset neurodegenerative disorders, such as Parkinson’s disease. The neural tube develops from its precursor tissue, the neural plate, in a patterning process that is determined by compartmentalization into morphogenetic units, the action of local signaling centers and a well-defined and locally restricted expression of genes and their interactions. While public databases provide gene expression data with spatio-temporal resolution, they usually neglect the genetic interactions that govern neural development. Here, we introduce Mouse IDGenes, a reference database for genetic interactions in the developing mouse brain. The database is highly curated and offers detailed information about gene expressions and the genetic interactions at the developing mid-/hindbrain boundary. To showcase the predictive power of interaction data, we infer new Wnt/β-catenin target genes by machine learning and validate one of them experimentally. The database is updated regularly. Moreover, it can easily be extended by the research community. Mouse IDGenes will contribute as an important resource to the research on mouse brain development, not exclusively by offering data retrieval, but also by allowing data input.

**Database URL:** http://mouseidgenes.helmholtz-muenchen.de.

## Introduction

Brain formation during vertebrate development is a complex process that has been studied for decades. The understanding of neuronal development is a prerequisite for the fight not only against neurodegenerative diseases, e.g. Parkinson’s disease, but also toward neuropsychiatric disorders in particular schizophrenia, autism disorders and drug addiction.

The emergence of the neural tube from the neural plate and the patterning of these structures along their anterior– posterior, dorsal–ventral and medial–lateral axes are fundamental processes during vertebrate neural development. The formation of forebrain, midbrain, hindbrain and spinal cord is determined by well-defined and locally restricted expression of genes and their gene regulatory networks (1). Whereas the patterning of the dorso–ventral axis depends on the relative amounts of dorsalizing and ventralizing factors such as the bone morphogenetic protein (BMP) and Sonic hedgehog (Shh), respectively, the patterning along the anterior–posterior axis is usually accomplished by local signaling centers such as the isthmic organizer (IsO) (2). The IsO, which is necessary and sufficient for the development of mesencephalic and metencephalic structures, is located at the boundary between midbrain and hindbrain and is, therefore, also referred to as the mid-/hindbrain boundary (MHB). The IsO also controls the generation of clinically highly relevant cell populations such as the ventral midbrain dopaminergic neurons, which are involved in Parkinson’s disease, schizophrenia and drug addiction, or the rostral hindbrain serotonergic neurons, which take part in mood disorders and depression. Therefore, the MHB or IsO is not only of developmental importance but also of high clinical relevance and thus subject of intense investigations (1–10). Up to now, four stages are thought to be necessary for the development of the MHB: (i) positioning and establishment, (ii) induction, (iii) maintenance and (iv) morphogenesis (1, 2, 7).

Positioning of the future MHB is almost exclusively achieved by the cross-inhibitory interaction of orthodenticle homolog 2 (Otx2) and gastrulation brain homeobox 2 (Gbx2), two transcription factors initially expressed in the anterior and posterior part of the developing embryo, respectively. The inductive mechanism for these two and other factors of the IsO in the neural plate are still unknown. Wingless-type MMTV integration site family member 1 (Wnt1) and fibroblast growth factor 8 (Fgf8) are two factors secreted from the anterior and posterior region of the MHB, respectively. Wnt1 is required for the maintenance of the MHB, and Fgf8 is necessary for the patterning of the midbrain and rostral hindbrain. The engrailed genes En1 and En2 as well as the paired box transcription factors Pax2 and Pax5 act up- and downstream of Wnt1 and Fgf8, mediating their maintenance as well as patterning function at the MHB (1, 2).

Advances in understanding the signaling cascades that give rise to distinct neuronal populations open new prospects for clinical therapies, like stem cell–based treatments. On the other hand, it allows clinicians to classify malformations of the brain more precisely, as with the help of embryology and genetics the major categories of a classification are the causative genes and their pathways and not exclusively the clinical phenotype (8–10).

The gene expression in neural development has been subject to many large-scale studies, and the results were stored in publically available databases. The most important of these resources were recently reviewed (11) and in the following a few will be exemplified. The mouse gene expression database developed by Mouse Genome Informatics (MGI) is a community resource for gene expression information from the laboratory mouse (12). It is designed as a database to collect and integrate raw expression data from a wide range of sources, such as RNA *in* *situ* hybridization, immunohistochemistry, western blots, northern blots and RT-PCR. Other databases focus on *in* *situ* hybridization data: The Allen Developing Mouse Brain Atlas [part of the Allen Brain Atlas (13)], GenePaint.org (14) and the e-Mouse Atlas of Gene Expression (15). Further, there is the Mouse Atlas of Gene Expression, which collects expression data based on serial analysis of gene expression (SAGE) (16). SAGE is more quantitative than *in* *situ* hybridization but lacks the high spatial resolution. While experimental methods like western and northern blots as well as RT-PCR are not suitable to derive information about exact spatial gene expression (e.g. within single cell populations), the main disadvantage of immunohistochemistry often represents the lack of a functional antibody. Notably, for many of the genes expressed at the MHB suitable antibodies are not available.

To facilitate, for example, dynamic modeling approaches, which necessitate a priori knowledge, i.e. highly curated data, a comprehensive collection of known genetic interactions containing spatial and temporal information is essential (17). These dynamic approaches are means to provide valuable insight into biological problems (17–20). Another interesting field for which integration of interaction and expression data was applied represents the prediction of new transcription factor binding sites (TFBSs) by using statistical models (21, 22). Although, plenty of gene expression data for the developing mouse brain are publicly accessible, interaction databases such as STRING (23), IntAct (24) or BioGRID (25) do not provide high spatial resolution on a developmental time scale. Thus, additional information about the interaction type [IEXP (inferred from experiment and/or expression pattern), ‘direct’, ‘direct signaling’, ‘indirect’, ‘indirect signaling’ or ‘maintenance’, which means to keep a gene active/inactive if it was already turned on/off] and mode (i.e. activation or repression) is not yet available for the specific genetic interaction network at the MHB and other brain regions.

We thus developed Mouse IDGenes, which represents a manually curated reference database for genetic interactions in the developing mouse brain focusing on the MHB, but with the possibility to add gene expression and interaction data of the central nervous system (CNS) with the help of a graphical user interface. The freely available database can be accessed via a Web interface through the URL http://mouseidgenes.helmholtz-muenchen.de. The Web interface offers detailed information about the expression of genes and their genetic interactions in the developing mid-/hindbrain region. Stored data were already used in part to understand regulatory gene interactions on the systems level (26, 27). Therefore, the resource provides the possibility for the simulation of the processes occurring at the MHB, which is a unique feature of the presented Web page. The Mouse IDGenes project is conceived for a continuous expansion of stored gene expression and interaction data. Users can enter new data in the database via the Web interface. Currently, 89 spatio-temporally resolved *in vivo* gene expression data sets and 145 genetic interaction data sets from 154 original publications assigned to different anatomical regions at mouse embryonic developmental stages E8.5, E10.5 and E12.5 (Theiler Stages 13, 17 and 20) are available from the database.

## Brain regionalization model

**Figure 1.**
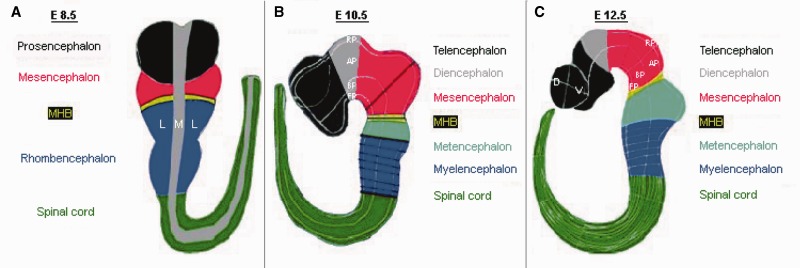
CNS regionalization of the mouse embryo at different developmental stages as used for the database structure. (**A**) Developmental stage E8.5: The whole embryo is divided into five regions along the anterior–posterior axis: prosencephalon, mesencephalon, MHB, rhombencephalon, spinal cord; along the medial–lateral axis the embryo is divided into medial and lateral, and the region in between is considered as mediolateral boundary. (**B**) Developmental stage E10.5: The embryo regionalization along the anterior–posterior and along the dorsal–ventral (previous medial–lateral) axis is as follows: telencephalon, diencephalon, mesencephalon, MHB, metencephalon (r1), myelencephalon (r2-r8) and spinal cord; for all CNS regions, the new regionalization along the dorsal–ventral axis is RP, AP, ABB, BP and FP. (**C**) Developmental stage E12.5: The mouse embryo is regionalized along the anterior–posterior axis as follows: telencephalon (anterior, posterior), diencephalon (anterior hypothalamus, posterior hypothalamus, prethalamus, thalamus), mesencephalon (anterior, posterior), MHB (anterior, posterior), metencephalon (r1), myelencephalon (r2-r8) and spinal cord (cervical, thoracic, lumbar, sacral, caudal); the telencephalon, diencephalon and MHB are subdivided along the dorsal–ventral axis into RP, AP, ABB, BP and FP; the mesencephalon, metencephalon and myelencephalon are subdivided into dorsal and ventral regions and/or neuronal populations along the dorsal–ventral axis; the spinal cord is subdivided into roof plate, dl1, dl2, dl3, dl4, dl5, dl6, v0, v1, v2, v3, v4, mn and floor plate, where dl1 to dl6 describe the dorsal interneurons, and v0 to v3 and mn denote the ventral interneurons (not shown).

We introduced a CNS regionalization model, which covers developmental stages E8.5, E10.5 and E12.5 (Theiler Stages 13, 17, and 20) representing three crucial stages in the development of the murine MHB and mid-/hindbrain region (Figure 1, Supplementary Table S1). The development of the MHB and of the mid-/hindbrain region initiates after gastrulation is finished. At E10.5, the establishment of the IsO at the MHB is completed, and its function at this stage is well characterized, meaning that most of the known interactions are taking place at this time point. The neural tube already exists at this stage, whereas specific neuronal populations have not developed yet. These neuronal populations, however, are first identifiable at around E12.5. Our model about the mouse anatomy is based on data reviewed from literature (28–32) and the MGI database (http://www.informatics.jax.org). To comply with the Edinburgh Mouse Atlas Project (EMAP), ontologies of mouse developmental anatomy, which provides a standard nomenclature for the description of normal and mutant mouse anatomy (33) and, therefore, to allow reusability of the data, we provide EMAP identifiers and descriptions for the defined brain regions (Supplementary Table S1). Because EMAP ontologies were recently updated to the EMAPA ontology (34), we mapped the presented brain regions also to these identifiers (Supplementary Table S1). The regionalization model was kept as general as possible, but as exact as necessary and covers initially the anterior–posterior compartmentalization of the neural tube into the brain vesicles and spinal cord. These brain vesicles correspond to the regions of the brain, which have already developed at a given developmental stage. Within these compartments, we further divided the regions on the anterior–posterior (i.e. longitudinal) axis into lateral and medial or dorsal and ventral parts depending on the corresponding developmental stage. Therefore, we developed a tripartition of a respective CNS region, which has the general structure of vesicle→anterior–posterior localization→medial–lateral or dorsal–ventral localization (column ‘Vesicle’, ‘Structure AP’, ‘Structure ML/DV’ in Supplementary Table S1, respectively). Whenever no further division was made, the description ‘all’ was used.

The CNS regions defined at developmental stage E8.5 are the prosencephalon, mesencephalon, MHB, rhombencephalon and spinal cord. Because the 2D neural plate has not yet folded up to give rise to the 3D neural tube, we only defined an anterior–posterior axis and a medial– lateral axis at this stage. At E10.5, the 3D neural tube undergoes further regionalizations on the anterior– posterior axis as well as on the dorso–ventral (previous medial–lateral) axis. For all CNS regions, the new regionalization on the dorso–ventral axis is roof plate (RP), alar plate (AP), alar basal boundary (ABB), basal plate (BP) and floor plate (FP). The prosencephalon separates along the anterior–posterior axis into the telencephalon and the diencephalon, the developing mesencephalon separates into anterior and posterior, the rhombencephalon splits into met- and myelencephalon and further into eight rhombomeres. The metencephalon was defined as consisting only of rhombomere one (r1).

Because these subdivisions are not defined as sharply as before and distinct neuronal populations have already arisen or are arising in the mid-/hindbrain region of the E12.5 mouse embryo, we additionally defined individual neuronal populations and new subdivisions of the regions (compartments) tel-, di-, mesencephalon, MHB, met- and myelencephalon and also spinal cord. At this stage, the telencephalon as well as the diencephalon show additional subdivisions along the dorsal–ventral axis, and the mesencephalon is subdivided into regions or neuronal populations (or both) also along the dorsal–ventral axis. While at E12.5 the MHB is still subdivided along the anterior– posterior and dorsal–ventral axis, in the met- and myelencephalon as well as spinal cord, dorsal and ventral regions or neuronal populations (or both) are defined along the dorsal–ventral axis. Along the anterior–posterior axis, the diencephalon is now subdivided into anterior hypothalamus, posterior hypothalamus, prethalamus and thalamus. In addition, at E12.5, the developing spinal cord is subdivided into five anterior–posterior regions, namely cervical, thoracic, lumbar, sacral and caudal.

## Database and Web page

Mouse IDGenes was implemented as a relational database using PostgreSQL (http://www.postgresql.org). Gene expression and interaction data were manually extracted from literature and stored in the database including references. Currently, the database contains 89 expression data sets and 145 genetic interactions. Genetic interactions as well as expression data sets are assigned to different anatomical regions at the mouse embryonic developmental stages E8.5, E10.5 and E12.5, as described before. To assess the quality of our data, we compared all interactions with the STRING database (v 9.1), a comprehensive resource of protein–protein interactions (23). All interactions but one in the current Mouse IDGenes data set are present in STRING with a high-confidence score (>0.7). The database was made accessible online through a Java Web interface, which was implemented using the Java Servlet class and runs on an Apache Tomcat Server. The Web interface allows the user to browse the Mouse IDGenes database for expression data and genetic interactions. By subscribing to a mailing list, it facilitates communication with other users of the database, as well as with the developers of the database and the Web page. Data can be retrieved in a legible PDF file format and as tab-delimited flat files (by navigating to the ‘Download’ tab on the Web page). By allowing external user an easy input (using the ‘Input Data’ tab on the Web page), which will be curated and evaluated by the authors, Mouse IDGenes will be continuously updated and thereby stay an up-to-date research tool.

### Detailed search

Navigation on the Mouse IDGenes Web page by the ‘Detailed Search’ tab allows users to search for gene expression and interaction data in specific regions of the embryonic mouse CNS at mouse embryonic developmental stages E8.5, E10.5 and E12.5 (radio button: ‘display genes according to the chosen region’). CNS regions and developmental stages (according to Supplementary Table S1) can be selected with the help of combo boxes. Having set a specific developmental stage and an anatomical structure, users are further able to analyze whether a specific gene of interest is expressed during that specific developmental stage in the selected anatomical structure.

### Search for interactions

On the ‘Search for Interactions’ tab, the Mouse IDGenes Web page allows users to search for specific interactions between two genes of interest and for all interactions in which one specific gene of interest is involved (by the ‘Search for specific interactions’ button). Information about the region and at which stage the specific interaction takes place in the embryonic mouse CNS can be retrieved.

Users can also search whether two genes of interest display an overlapping gene expression (by the ‘Search for overlapping gene expressions’ button).

By selecting ‘all’ in either both or only one of the gene selection boxes, users can search for all stored interactions either of the whole database or in which a specific gene of interest is involved.

The displayed interactions follow an overall scheme consisting of the attributes ‘effect’, ‘type’ and ‘name’ for a genetic interaction. The attribute ‘effect’ can be either activation, i.e. turning on gene expression, or repression, which is defined as shutting down gene expression. The attribute ‘type’ of an interaction is defined by the following six values:

– direct: We define a direct interaction in case interaction partner 1 binds directly to the promoter of interaction partner 2.
– direct signaling: In case of a ligand that activates a signaling cascade or any other component of a signaling cascade that does not directly interact with (or binds to) the promoter of a target gene of this signaling pathway, a direct signaling interaction refers to the activation/repression of a direct target gene of this signaling pathway.
– indirect: Interaction partner 1 does not bind directly to the promoter of interaction partner 2, and signaling or genetic interaction cascades have to occur between the two interaction partners.
– indirect signaling: In case of a ligand that activates a signaling cascade or any other component of a signaling cascade that does not directly interact with (or binds to) the promoter of a target gene of this signaling pathway, an indirect signaling interaction refers to the activation/repression of an indirect target gene by a direct target gene of this signaling pathway.
– maintenance: Interaction partner 1 is not required to activate (i.e. turn on) or to repress (i.e. turn off) the promoter (or expression) of interaction partner 2, but to keep this promoter (or expression) activated (‘on’) or repressed (‘off’) over (a longer period of) time.
– IEXP: Inferred from experiment (e.g. loss of function/gain of function) and/or expression pattern

Currently, the database contains these general interaction schemes: direct activation, direct signaling activation, IEXP activation, maintenance activation, indirect activation, indirect signaling activation, direct repression, direct signaling repression, maintenance repression, IEXP repression, indirect repression and indirect signaling repression. The attribute ‘name’ of an interaction, which is displayed on the Mouse IDGenes Web page, is composed of the official gene symbol according to MGI of interaction partner 1, followed by an arrow symbol from the third column of Table 1 and finally the gene symbol of interaction partner 2.

**Table 1.** General scheme for interactions used at the Mouse IDGenes Web page is listed; interactions are divided into a type, which can be ‘direct’, ‘direct signaling’, ‘indirect’, ‘indirect signaling’, ‘IEXP’ and ‘maintenance’, as well as an effect, namely ‘activation’ or ‘repression’ of gene expression

Interaction type	Interaction effect	Symbol
Direct	Activation	- >
Direct signalling	Activation	- >
Indirect	Activation	- - >
Indirect signalling	Activation	- - >
IEXP	Activation	- >
Maintenance	Activation	- >
Direct	Repression	- |
Direct signalling	Repression	- |
Indirect	Repression	- - |
Indirect signalling	Repression	- - |
IEXP	Repression	- |
Maintenance	Repression	- |

Direct: a transcription factor (interaction partner 1) directly binds to the promoter of a gene (interaction partner 2); direct signaling: an activation/repression of a direct target gene of a specific pathway initiated by a ligand that activates the signaling cascade of this pathway; indirect: interaction partner 1 does not bind directly to the promoter of interaction partner 2, and signaling or genetic interaction cascades have to occur between the two interaction partners; indirect signaling: an activation/repression of an indirect target gene through a direct target gene of a specific pathway initiated by a ligand that activates the signaling cascade of this pathway; IEXP: inferred from experiment (e.g. loss of function/gain of function) and/or expression pattern; maintenance: interaction partner 1 is not required to activate (i.e. turn on) or to repress (i.e. turn off) the promoter (or expression) of interaction partner 2 but to keep this promoter (or expression) activated (‘on’) or repressed (‘off’) over (a longer) time.

As pointed out before, the maintenance activation occurs over a longer period to cause a downstream effect. Time-wise, such an interaction can occur over several developmental stages as, for example, is the case of the development of midbrain dopaminergic (mDA) neurons. There, initially a Wnt1-regulated network together with a Shh-controlled genetic cascade establishes the mDA progenitor domain, by maintaining Otx2 expression in the ventral midbrain (35). Another example is Lmx1b, which is known to be necessary for the initiation of Fgf8 expression and for the maintenance of several other genes including Engrailed 1 (En1), En2 and Wnt1 (3). Lmx1b, therefore, falls into the interaction scheme ‘maintenance activation’ for En1, En2 and Wnt1. On our Web page, these interactions are depicted in the following way: Lmx1b → En1, Lmx1b → En2 and Lmx1b →Wnt1, meaning that Lmx1b keeps the expression of En1, En2 and Wnt1, respectively, on overtime.

Furthermore, cooperative interactions, i.e. interactions with more than two interaction partners, can be stored in the database, for example, if two transcription factors or one transcription factor and another cofactor will bind on the promoter of a target gene, i.e. interaction partner 2.

### Data input

**Figure 2.**
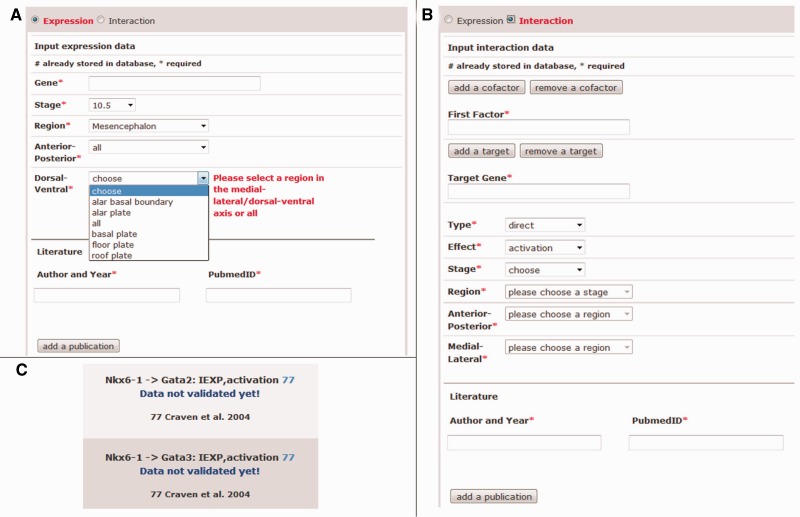
Dialogue of the data input and output of not yet validated data as found on the Mouse IDGenes Web interface. Input dialogue for (**A**) expression data and (**B**) interaction data. (**C**) The output of not yet validated data.

One of the core functions of Mouse IDGenes is the possibility for data input by the user. This feature allows the permanent update of data by the respective experts in the field of developmental neurosciences. To input new gene expression and interaction data into the database, the user is asked to maintain the overall CNS regionalization scheme (according to Supplementary Table S1), by choosing and subsequently storing specifications given by combo boxes, which follow our CNS model (Figure 2A). The database so far has stored data exclusively about the developmental stages E8.5 to E12.5, as these are the crucial stages in the establishment of the MHB and development of the mid-/hindbrain region, which is the authors’ main research interest. To maintain the high degree of curation in the current database, it is necessary to also introduce at least one relevant publication as well as the corresponding PubMed ID.

Users can input gene expression data as well as interaction data into the database by selecting the respective type of data on the Web page. All fields that are mandatory for data input as well as storage in the database are labeled by an asterisk (Figure 2A and B), which ensures the completeness of expression and interaction data sets. In addition, it is automatically controlled from the PubMed abstract by using the link to PubMed whether the given year of the publication and the entered PubMed ID are consistent (compare with the fields ‘Author and Year’ and ‘PubmedID’ in Figure 2A and B); otherwise, a warning message is displayed, and storage of the data is prevented. Further, gene symbols from MGI are auto-completed after typing some letters in the field ‘Gene’ (Figure 2A) or ‘First Factor’ or ‘Target Gene’ (Figure 2B), and they are internally stored via MGI identifiers. The use of predefined lists by combo boxes for the selection of e.g. a specific brain region and/or the interaction type helps to comply with our brain regionalization model as well as the model for genetic interactions and therefore ensures data consistency. In case of extending the database for an interaction, it is possible to input also cofactors and more targets of an interaction (Figure 2B). After completion of the data set, the user is asked to review the input before final submission to the database. Constraints in the PostgreSQL database prevent data from being duplicated when stored.

To control for incorrect input, the data will be regularly curated by the authors. Before curation, new data will be distinguishable on the Web page from already curated data by labeling the not yet validated gene expression or interaction data on the Web page (Figure 2C).

Confirmation of new data is performed by

– reading the given publications,
– comparing the indicated gene expression and/or genetic interaction,
– comparing the developmental stage as well as the brain regions with the data entered into the Mouse IDGenes database.

A mailing list has been set up for users to discuss their plan to submit new data.

After verification of new data, the flag ‘Data not validated yet!’ is removed from the database and the Web page; otherwise, if the data could not be verified, they are deleted from the database.

### Output

**Figure 3.**
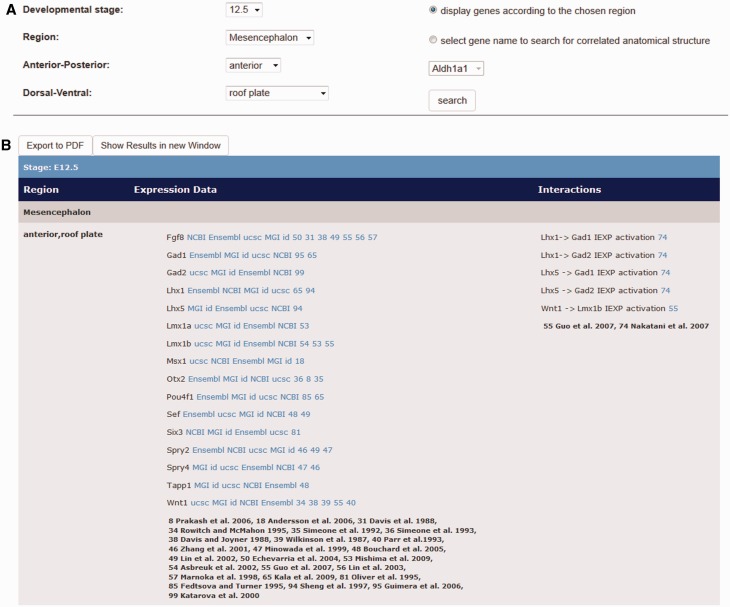
Output window of expression data and interaction data by the use of the ‘Detailed Search’ option on the Mouse IDGenes Web page. (**A**) Search dialogue on the menu item ‘Detailed Search’. In this example, expression and interaction data for the roof plate of the anterior mesencephalon at embryonic day 12.5 are requested. (**B**) Output for gene expression data as well as interaction data for the request according to (A).

The output of a specific search either in one of the gene expression or the interaction menu items appears on the same page underneath the user selection (Figure 3B), organized in a table. In case of the gene expression data (radio button: ‘display genes according to the chosen region’ (Figure 3A) in the ‘Detailed Search’ menu item), the output table indicates the developmental stage the user is looking at. The output further shows the specific subdivision of the chosen anatomical area and which genes are expressed there in the column ‘Region’. Additionally, links to the public databases NCBI, MGI, UCSC and Ensembl are given in the column ‘Expression Data’, where further general information about a displayed gene can be retrieved. Most importantly, links to literature references are indicated as evidence of gene expression information. The last column ‘Interactions’ of the output shows the interaction data, which can be retrieved for the actual brain region at the actual developmental stage with the corresponding literature references.

## Application of the database

### Prediction of a new Wnt1 target based on Mouse IDGenes

To demonstrate the usefulness of the Mouse IDGenes database for other research applications, we chose an example from our own scientific interests focused on the role of the Wnt signaling pathway in the development of the mid-/hindbrain region and of neuronal populations located in this region, such as the ventral midbrain dopaminergic neurons. In this context, the Wnt signaling pathway plays a crucial role because it participates in the regulation of regional patterning, cell cycle, cell fate specification, cell differentiation and cell survival. It is also involved in various human diseases (36).

The manually curated, and thus, highly reliable data set of Mouse IDGenes provides an ideal basis to further analyze, for example, the complex gene regulatory network at the MHB and in the ventral midbrain in which Wnt1/β-catenin signaling has so far been implicated (37, 38). We used the Mouse IDGenes database to predict novel direct or indirect targets of the Wnt1/β-catenin signaling pathway that might be involved in the development of the mid-/hindbrain region and of associated neuronal populations and validated our results experimentally.

**Figure 4.**
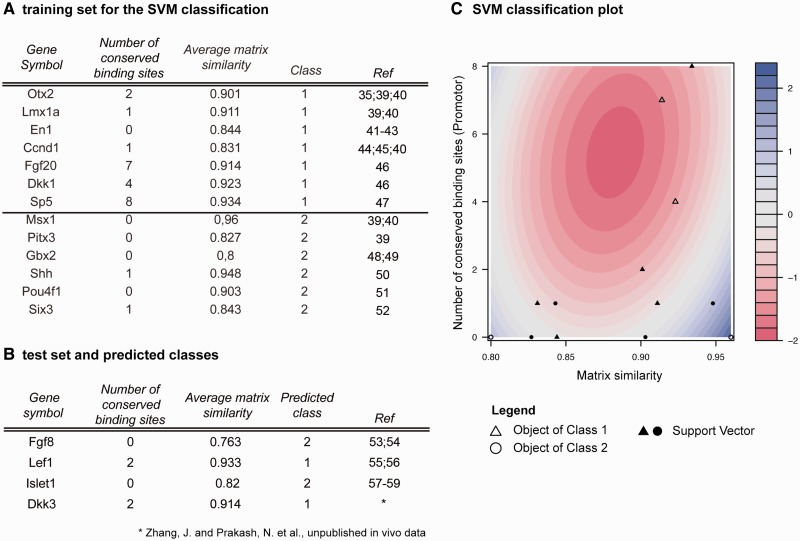
Training and test data for SVM classification. (**A**) Training data containing experimentally validated Wnt1 target genes. Genes were extracted from Mouse IDGenes, and number of conserved binding sites and average matrix similarity were computed with Genomatix MatInspector. Class 1 contains Wnt1 targets of the type ‘direct signaling’ (i.e. direct Lef1/Tcf target genes), whereas class 2 includes Wnt1 targets of the type ‘indirect signaling’. (**B**) Result of classification of selected genes from the test set. Direct Lef1/Tcf targets have more conserved binding sites and a higher average matrix similarity. (**C**) SVM classification (contour) plot showing training data. Filled objects indicate support vectors, blank objects remaining data points. Red color indicates decision values of class 1 (i.e. direct Lef1/Tcf binding), while blue color indicates decision values of class 2 (indirect Lef1/Tcf binding).

**Figure 5.**
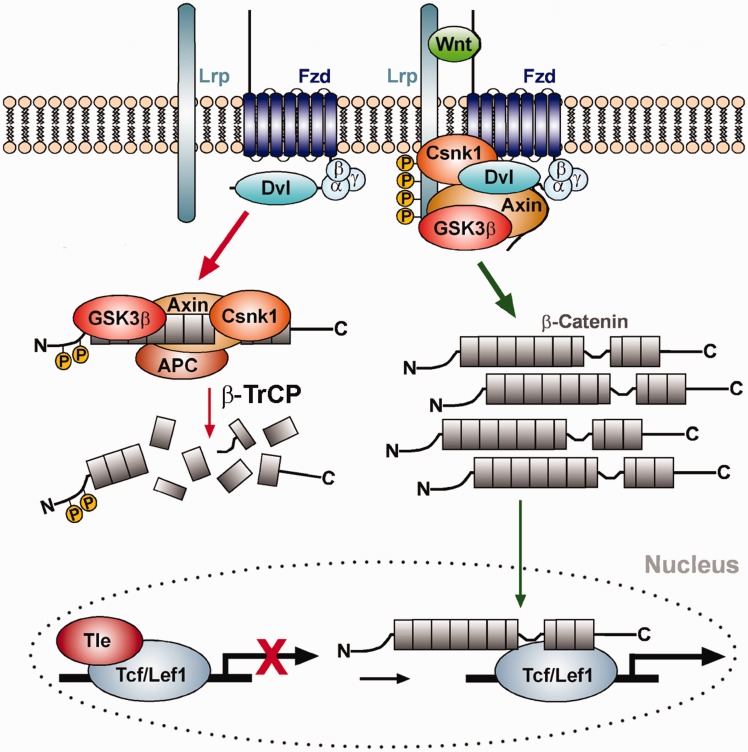
The Wnt/β-catenin signaling pathway. Left (red arrows): In the absence of Wnt ligand, β-catenin is bound by the destruction complex consisting of the scaffolding proteins Axin and Adenomatosis polyposis coli (APC), and the protein kinases Glycogen synthase kinase 3 beta (GSK3β) and Casein kinase I (Csnk1), and sequentially phosphorylated by these kinases. Phosphorylated β-catenin binds to and is ubiquitinated by the E3 ubiquitin ligase β-TrCP, thereby targeting it for proteasomal degradation. In the absence of Wnt ligand, lymphoid enhancer binding factor 1 (Lef1) or T cell-specific (TCF) transcription factors are bound to the promoters of Wnt target genes in the cell nucleus together with co-repressors of the Groucho/transducin-like enhancer of split (Tle) family proteins, thereby repressing their expression. Right (green arrows): On binding of Wnt ligand to the Frizzled (Fzd) receptor and low-density lipoprotein receptor-related protein (Lrp) co-receptor complex, Axin and GSK3β are recruited to the cell membrane via Dishevelled (Dvl) and the destruction complex falls apart. Unphosphorylated β-catenin accumulates in the cytosol and translocates into the nucleus, where it binds to the Lef1/TCF transcription factors and activates Wnt target genes by displacing the co-repressors and recruiting co-activators to this complex. Properties of Lef1/Tcf binding sites in the promoters of known Wnt target genes, i.e. the number of conserved Lef1/Tcf binding sites as well as the averaged matrix similarity, were used to train a classifier and to predict direct or indirect interactions of potentially new target genes and Lef1/Tcf transcription factors in the Wnt/β-catenin signaling pathway.

We obtained known targets with the interaction type ‘direct signaling’ and ‘indirect signaling’ of the Wnt1/β-catenin pathway from the Mouse IDGenes Web page by choosing Wnt1 as search term in the ‘Search for Interactions’ menu as well as from literature searches (Figure 4A) (35, 39–52). One hallmark of the Wnt1/β-catenin signaling pathway is the stabilization and nuclear translocation of cytoplasmic β-catenin (Figure 5). In the absence of a Wnt1 signal, the Lef1/Tcf transcription factors are bound to the promoter regions of the direct Wnt1 target genes together with other co-repressors, thereby inhibiting the activation of these genes. In the presence of a Wnt1 signal, the replacement of these co-repressors and binding of β-catenin to the Lef1/Tcf transcription factors activates the transcription of the direct Wnt1 target genes. The frequent presence of, in particular, evolutionary conserved Lef1/Tcf TFBSs in the promoter region of a gene is therefore indicative that this gene might be a direct target gene of the Wnt1/β-catenin signaling pathway. An indirect target gene of the Wnt1/β-catenin pathway was defined as a gene that is upregulated on Wnt1/β-catenin signaling activity but is not directly bound by β-catenin and Lef1/Tcf transcription factors and thus requires another mediator, i.e. an intermediate gene regulatory step.

We performed an *in silico* promoter analysis for Wnt1 target genes of the interaction type ‘direct signaling’ and ‘indirect signaling’ in the training data set (Figure 4A) as well as in the test set (Figure 4B) consisting of interesting target genes for which the interaction type is not yet known in the CNS (53–59). The MatInspector (Genomatix) program was used to identify Lef1/Tcf binding sites in the promoter sequences of these target genes with help of predefined position weight matrices (PWMs) (60). For classification of the interaction type, we assigned ‘direct signaling’ target genes of Wnt1/β-catenin pathway to class 1 and ‘indirect signaling’ targets to class 2. We computed two parameters for each gene: (i) the number of evolutionary conserved Lef1/Tcf TFBSs (V$LEFF). Orthologous promoter sequences from human, chimp, mouse, rat, dog, horse, cow and opossum were taken into account, and only binding sites present in at least two species were considered. (ii) The average matrix similarity of all Lef1/Tcf binding sites in the promoter, a score indicating the similarity of a predicted binding site with the consensus matrix of the TF (60, 61). It lies in [0, 1] and reaches 1 only if the predicted sequence corresponds to the most conserved nucleotide at each position. While the matrix similarity allows the assessment of the structural quality of a binding site, the number of conserved binding sites supports the possibility that these binding sites might be functionally relevant (62). It is assumed that in case of direct interactions the matrix similarity is higher and conserved binding sites are more frequent than in case of indirect interactions.

We trained a support vector machine (SVM) with ‘direct signaling’ targets (class 1) and ‘indirect signaling’ targets (class 2) using both parameters (Figure 4C). Classification via SVM has been successfully used not only for feature selection of microarray data (63–65) but also to integrate expression as well as genomic data, e.g. evolutionary conservation or binding site clusters for the improvement of TFBS prediction (21, 22). The statistical model was calculated with help of the ksvm function from the kernlab package in R statistical software by using a polynomial kernel matrix similarity (degree = 2), a cost parameter C = 1 and a 13-fold cross validation (leave-ne-out cross validation). Class 1 consists of Otx2, Lmx1a, En1, Ccnd1, Dkk1 and Sp5 and class 2 consists of Msx1, Pitx3, Gbx2, Shh, Pou4f1 (Brn3a) and Six3 (Figure 4A). Additionally, Fgf20 was included in class 1. Interaction data for this gene cannot be retrieved from Mouse IDGenes, as expression of Fgf20 in the mouse neural tube starts only after E12.5 (66). We assigned En1 to class 1 because it was shown that En1 expression under the control of the Wnt1 enhancer in mice rescues the Wnt1-/- mid-/hindbrain phenotype (41, 42), thus indicating that En1 is the downstream target of Wnt1 signaling in mid-/hindbrain development, and because a direct interaction of the promoter of the homologous gene engrailed-2 (En2) with the Lef1/Tcf transcription factor was observed in the frog (43). For our statistical model, we obtained a training error of 7.69% and a cross-validation error of 46.15%.

To further elucidate the Wnt1-controlled gene regulatory network at the MHB, we applied SVM prediction by using the number of conserved Lef1/Tcf binding sites and the average matrix similarity in the promoters of four interesting genes, Fgf8, Lef1, Islet1 and Dkk3, representing the test set (from Figure 4B) to predict whether they are directly or indirectly activated by the Wnt1/β-catenin pathway. For these four genes, it is not known whether they are direct or indirect targets of Wnt1 in neural tissues, but it was observed that Lef1 and Dkk3 are co-expressed with Wnt1 in the midbrain (Götz, S. et al., unpublished data), whereas Fgf8 and Islet1 are not co-expressed with Wnt1 but depend on Wnt1 expression in the mid-/hindbrain region (53, 54, 58, 59). Using the SVM on the test set (Figure 4B), our analysis predicts that Lef1 and Dkk3 are direct targets of Lef1/Tcf-mediated Wnt1/β-catenin signaling, whereas Fgf8 and Islet1 are not direct target genes of this signaling pathway and interact indirectly with the Wnt1 signaling cascade. Fgf8 and Islet1 therefore would represent Wnt1/β-catenin target genes that are most likely activated by other genes that in turn are activated by Wnt1/β-catenin signaling. Direct binding of Lef1/Tcf transcription factors to the Lef1 promoter was shown in colon cancer/lymphocytes (55) as well as in HEK 293 cells (56), which is in accordance with our prediction. A direct interaction of Lef1/Tcf transcription factors with a larger promoter region of the Islet1 gene was shown in embryonic heart tissue but not in neural tissues (57). However, direct activation of Fgf8 and Islet1 in the CNS by Lef1/Tcf binding sites was up to now never observed although it was inferred from mutant mouse embryo analyses (53, 54, 58, 59).

### Experimental validation of the predicted Wnt1 target gene Dkk3

To show the predictive power of our approach, we experimentally validated the so far unknown Wnt1 target gene Dkk3 as a direct target gene of the Lef1/Tcf-mediated Wnt1/β-catenin signaling cascade *in vitro*.

**Figure 6.**
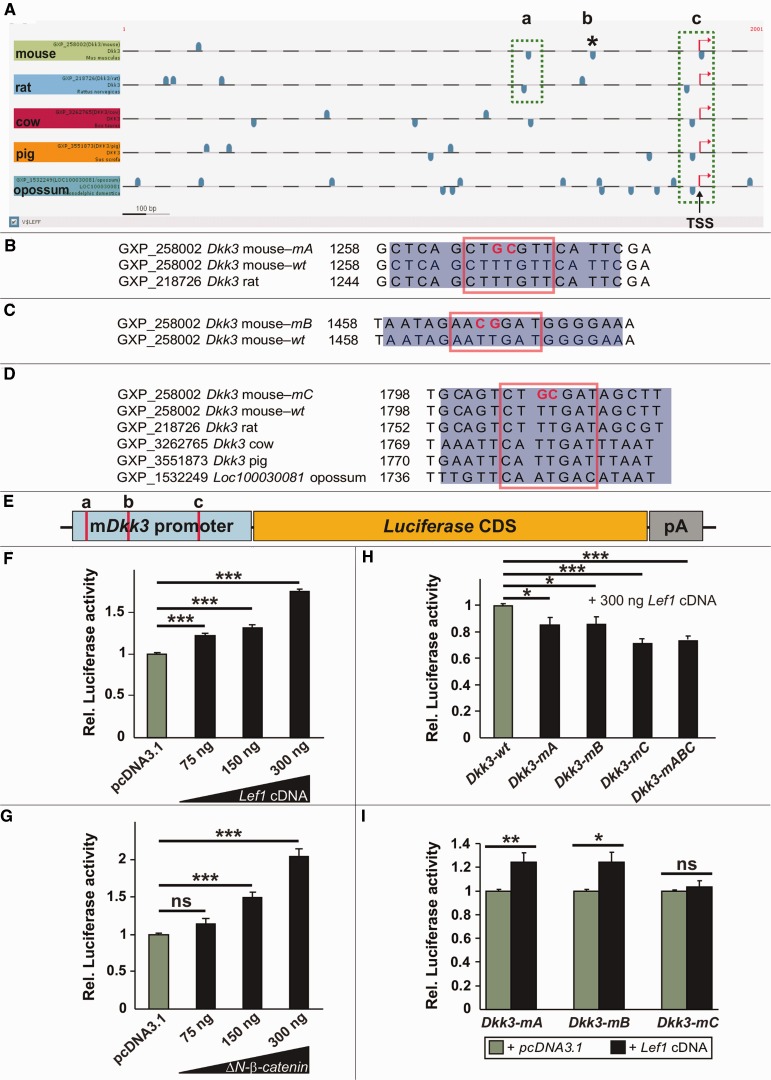
Mouse Dkk3 is a direct target gene of Lef1-mediated Wnt/β-catenin signaling. (**A**) Representation of the putative Dkk3 promoter (5′ proximal) regions from mouse, rat, cow, pig and opossum and of the predicted Lef1/Tcf binding sites on the sense (upper blue boxes) and antisense (lower blue boxes) strands within these Dkk3 promoter regions. The most conserved (by sequence similarity and position) and proximal (relative to the TSS) predicted Lef1/Tcf binding sites were designated as ‘a’ and ‘c’ (green dotted boxes). The Lef1/Tcf binding site ‘b’ is only predicted in the mouse Dkk3 promoter (asterisk). (**B–D**) Sequence alignments of the mutated (m) and wild-type (wt) Lef1/Tcf binding site ‘a’ in the mouse and rat Dkk3 promoter regions (B), ‘b’ in the mouse Dkk3 promoter region only (C) and ‘c’ in the mouse, rat, cow, pig and opossum Dkk3 promoter regions (D). The blue rectangles delimit the sequence, and the red boxes frame the core sequence of the corresponding Lef1/Tcf binding site. The red bold letters indicate the mutagenized nucleotides in the corresponding Lef1/Tcf binding site core sequence. (**E**) Schematic drawing of the murine Dkk3 (mDkk3) promoter/luciferase reporter construct used in the following experiments, and of the approximate position of the three predicted and partly conserved proximal Lef1/Tcf binding sites within this promoter fragment (red bars). CDS, coding sequence; pA, polyadenylation signal. (**F–I**) Luciferase reporter assays in HEK293T cells using the wild-type and mutated mDkk3 promoter/reporter construct depicted in (E). (F) Co-transfection of increasing amounts of Lef1 cDNA led to a dose-dependent activation of the wild-type mDkk3 promoter relative to the ‘empty’ (pcDNA3.1) vector control. (Rel. luciferase activities: pcDNA3.1, 1.0 ± 0.01; 75 ng Lef1 cDNA, 1.21 ± 0.03; 150 ng Lef1 cDNA, 1.32 ± 0.035; 300 ng Lef1 cDNA, 1.74 ± 0.04). (G) Co-transfection of increasing amounts of a constitutively active β-catenin (ΔN-β-catenin) led to a dose-dependent activation of the wild-type mDkk3 promoter relative to the ‘empty’ (pcDNA3.1) vector control. (Rel. luciferase activities: pcDNA3.1, 1.0 ± 0.01; 75 ng ΔN-β-catenin, 1.14 ± 0.07; 150 ng ΔN-β-catenin, 1.49 ± 0.06; 300 ng ΔN-β-catenin, 2.04 ± 0.11). (H) Site-directed mutagenesis of single and of all three predicted Lef1/Tcf binding sites (‘a’, ‘b’, ‘c’, ‘abc’) within the mDkk3 promoter fragment (Dkk3-mA, Dkk3-mB, Dkk3-mC, Dkk3-mABC) resulted in a site-specific and significant decrease of luciferase activity relative to the wild-type mDkk3 promoter (Dkk3-wt) after co-transfection of 300 ng Lef1 cDNA. (Rel. luciferase activities: Dkk3-wt, 1.0 ± 0.01; Dkk3-mA, 0.85 ± 0.05; Dkk3-mB, 0.86 ± 0.06; Dkk3-mC, 0.71 ± 0.04; Dkk3-mABC, 0.73 ± 0.03). (I) Site-directed mutagenesis of the most conserved (across species) and proximal (relative to the TSS) Lef1/Tcf binding site ‘c’ in the mDkk3 promoter completely abolished the activation of this promoter after co-transfection of 300 ng Lef1 cDNA relative to the ‘empty’ vector control (pcDNA3.1). (Rel. luciferase activities: Dkk3-mA: pcDNA3.1, 1.0 ± 0.01; Lef1 cDNA, 1.24 ± 0.08; Dkk3-mB: pcDNA3.1, 1.0 ± 0.01; Lef1 cDNA, 1.24 ± 0.08; Dkk3-mC: pcDNA3.1, 1.0 ± 0.01; Lef1 cDNA, 1.04 ± 0.05). *P < 0.05; **P < 0.01; ***P < 0.001; ns, not significant.

To identify conserved Lef1/Tcf binding sites in ∼700 bp extended promoter region (as used for the SVM prediction) of the Dkk3 gene of five different mammalian species (mouse, rat, cow, pig and opossum), we applied the DiAlign TF program in the Genomatix software suite GEMS Launcher to evaluate the overall promoter similarity and to identify conserved Lef1/Tcf binding sites in these regions. For the alignment, we chose the five most conserved Dkk3 promoter sequences among 14 organisms from Genomatix homology group Hg3927. Four Lef1/Tcf binding sites were predicted in the putative mouse Dkk3 promoter, of which one [binding site ‘c’, the most proximal Lef1/Tcf binding site to the transcription start site (TSS)] was highly conserved among all five species (Figure 6A). To determine whether these predicted Lef1/Tcf binding sites control the Wnt1/β-catenin and Lef1/Tcf-mediated activation of the murine Dkk3 promoter, we cloned a 744-bp-long fragment of this promoter containing three of the four predicted Lef1/Tcf binding sites into a promoter-less luciferase reporter vector (Figure 6E). Co-transfection of increasing amounts of rat Lef1 complementary DNA (67) or a constitutively active β-catenin [ΔN-β-catenin, which mimics the activation of Wnt1 signaling, (68)] into ‘Wnt-responsive’ HEK293T cells [exhibiting a basal level of Wnt1/β-catenin signaling activity, Prakash, N. et al., unpublished data, (69)] led to a dose-dependent activation of luciferase expression mediated by this mouse Dkk3 (mDkk3) promoter fragment (Figure 6F and G), indicating that the promoter of the mDkk3 gene is a direct target of Lef1-mediated Wnt1/β-catenin signaling in this *in vitro* context. Additional *in vivo* evidence indicates that the murine Dkk3 gene is also a direct target of Lef1-mediated Wnt1/β-catenin signaling in the mouse ventral midbrain (Zhang, J. and Prakash, N., unpublished data).

To evaluate whether the predicted Lef1/Tcf binding sites in the murine Dkk3 promoter are functional, we mutagenized each of these binding sites either individually (mutation of a single Lef1/Tcf binding site) or altogether (mutation of all three Lef1/Tcf binding sites) within the mDkk3 promoter fragment such that they cannot be recognized by Lef1/Tcf transcription factors anymore (Figure 6B–E) (46). Site-directed mutagenesis of single or all three Lef1/Tcf binding sites in the mDkk3 promoter/reporter constructs revealed that

– luciferace activity was significantly decreased relative to the wild-type mDkk3 promoter after co-transfection of Lef1 cDNA (Figure 6H),
– the activation of the mDkk3 promoter/reporter construct carrying a mutagenized Lef1/Tcf binding site ‘c’ by Lef1 was completely abolished relative to the pcDNA3.1 control, in contrast to a still significant activation of the mDkk3 promoter/reporter constructs carrying a mutagenized Lef1/Tcf binding site ‘a’ or Lef1/Tcf binding site ‘b’ (Figure 6I).

This result strongly suggests that the most conserved (by position and sequence similarity among five mammalian species) and proximal (relative to the TSS) Lef1/Tcf binding site ‘c’ is the functionally most important of the three Lef1/Tcf binding sites for Lef1-mediated activation of the murine Dkk3 gene by Wnt1/β-catenin signaling.

Lef1/Tcf transcription factors were predicted to directly activate the mouse Dkk3 gene in the context of Wnt1/β-catenin signaling by our SVM analyses. Therefore, the experimental validation of Lef1/Tcf binding site ‘a’ and Lef1/Tcf binding site ‘c’ with a matrix similarity of 0.867 and 0.961, respectively, confirm the result of our SVM classification, indicating that direct Lef1/Tcf targets have in general more conserved binding sites than indirect targets (Figure 4B). Altogether, our experimental results indicated that the activation of the mouse Dkk3 gene is mediated at least in part by the predicted Lef1/Tcf binding sites in its promoter region and therefore highlight the importance and predictive power of a database combining both expression and interaction data.

## Future directions

So far, the Mouse IDGenes database offers spatially resolved and manually registered data about the developmental stages E8.5, E10.5 and E12.5 of the mouse mainly with gene expressions and interactions important for the development of the mid-/hindbrain region because it harbors important neuronal populations that are implicated in several neurodevelopmental human diseases (1, 2, 7). We aim to enlarge the data sets to more entries that would provide a good representation of the known gene expression patterns and interactions in the developing mouse CNS. This aim is already facilitated by allowing users to input data to the database. With this, we intend to attract experts in different fields of developmental neurosciences to update the existing platform so that Mouse IDGenes becomes the data source of choice, for experimental and *in silico* analyses related to gene expression and interaction data in the developing murine CNS. Additionally, a broader data set will lead to improvement of dynamic modeling projects and to more precise prediction methods (26, 27). As demonstrated, the genetic interaction data stored in the Mouse IDGenes database can be used for the prediction of Wnt target genes, but with this database in combination with publicly available PWMs (70), it is also possible to predict new targets of other signaling pathways (e.g. Shh, Fgf8 or BMPs), which are equally important for CNS development. With our mailing list, we seek to develop an open platform, which eases the communication between neuroscientists.

## Materials and methods

### Bioinformatics prediction of Lef1/Tcf binding sites in the promoter regions of Wnt/β-catenin target genes

To discriminate interactions of the type ‘direct signaling’, i.e. genes bound by Lef1/Tcf transcription factors activated by Wnt1 signaling from correlations of the type ‘indirect signaling’ and to predict the interaction type of new target genes, a support vector machine (SVM) was applied. The data matrix of the SVM is composed of two variables (columns) for each object (row), i.e. the frequency as well as the averaged matrix similarity of conserved TFBSs for each promoter sequence of a specific gene. The classification of the interaction type in training data set for each object (gene), meaning whether Lef1/Tcf transcription factor binds directly or indirectly to the promoter sequence, was derived from the Mouse IDGenes database. Promoter sequences for Wnt/β-catenin target genes were derived from the ElDorado genome database (Genomatix/Germany), versions 12-2010 and 08-2011. Orthologous promoter sequences from different mammalian species of the Genomatix homology group (human, chimp, mouse, rat, dog, cow, pig and opossum) were analyzed using the MatInspector program (with the Matrix Family Library Version 8.4) from Genomatix to identify potential Lef1/Tcf binding sites and to extract matrix similarities. Conserved binding sites were determined by using the DiAlign TF program (Genomatix) and by searching for common Lef1/Tcf sites occurring at the same position in (aligned) orthologous promoter sequences of each Wnt/β-catenin target gene. The length of the promoter regions used for the detection of Lef1/Tcf sites to derive the total number and the average matrix similarity of binding sites both included in the SVM algorithm were generally in the range of 600–1400 bp. In addition, a longer promoter region of mouse Dkk3 mRNA (with the Genbank identifier AK013054, firstly detected in whole body of 10- and 11-day-old mouse embryos) was defined as 1800 bp upstream, including the proximal region, and 200 bp downstream of the TSS. Dkk3 promoter sequences of 2000 bp length from five different mammalian species (mouse, rat, cow, pig and opossum) were analyzed with the MatInspector to predict Lef1/Tcf binding sites.

### Cloning of a mouse Dkk3 (mDkk3) promoter/reporter vector

A 744-bp fragment of the putative mDkk3 promoter (Entrez Gene ID: 50781; chromosome 7, strand: −, position: 112 158 266 to 112 159 009 bp, NCBI build 38) was amplified from C57BL/6 mouse genomic DNA by PCR using the forward primer 5′-*ctcgag *TGACCA G A T C C A G C T TGCA-3′ and reverse primer 5′-*aagctt *CCTC CTGA GG GTAG TTGAGA-3′ that included an *Xho*I and *Hind*III restriction site (underlined sequences in italics), respectively. The amplified fragment was cloned into the pCR®II TOPO TA vector (TOPO® TA Cloning® Kit, Life Technologies/Germany) and sequenced throughout its entire length (Sequiserve/Germany). The mDkk3 promoter fragment was excised from the pCR®II TOPO TA vector by *Xho*I/*Hind*III digestion and subcloned into an *Xho*I/*Hind*III-digested pGL3-Basic Vector (Promega/USA).

### Site-directed mutagenesis of the mDkk3 promoter fragment

Site-directed mutagenesis of the most conserved and proximal (relative to the TSS) Lef1/Tcf binding sites predicted in the 744-bp mDkk3 promoter fragment was done using the QuickChange Lightning Multi Site-Directed Mutagenesis Kit (Agilent Technologies/USA) according to the manufacturer’s instructions. Mutagenic primers were the following: Dkk3-mA: 5′-ccagcttgcagctcag*ct****gc****gtt*cattc gaa ttgggtg-3′; Dkk3-mB: 5′-gtccaagagatcccagtaatag*aa****cg****gat*gg ggaaa tagta aaggaa-3′; Dkk3-mC: 5′-ggtggtcctgcagt*ct****gc****ga t*agctttccgggac-3′ (mutagenized nucleotides in bold; core sequence of the corresponding Lef1/Tcf binding site in italics). Mutated promoter fragments were confirmed by sequencing (Sequiserve).

### Cell culture, transfections and luciferase reporter assays

HEK-293T cells were kept at 37°C and 5% CO_2_ in DMEM medium + 10% fetal calf serum/glutamine (Life Technologies). HEK-293T cells (1.25 × 10^5^ cells/well of a 24-well plate) were co-transfected with 300 ng/well pGL3-mDkk3 promoter/reporter vectors (wild-type and mutagenized sequences), 30 ng/well pRL-SV40 (as internal transfection control, Promega) and 150–225 ng/well pcDNA3.1 (Life Technologies) ‘empty’ vector, alone or together with 75 ng/well, 150 ng/well or 300 ng/well constitutively active ΔN-β-catenin (68) or rat Lef1 cDNA (67) using Lipofectamine 2000 (Life Technologies). The total amount of plasmid DNA transfected in each well was 630 ng. Cells were lysed in Passive Lysis Buffer (Promega) after 24 h, and Firefly and Renilla Luciferase luminescence were measured in a Centro LB 960 luminometer (Berthold Technologies/Germany) using the Dual-Luciferase® Reporter Assay System (Promega) according to the manufacturer’s instructions. Firefly luminescence was normalized against Renilla luminescence for each well. Assays were performed in triplicates, and data are derived from three independent experiments.

### Statistical analyses

All values shown are mean ± SEM. Statistical significance between groups was assessed by two-tailed independent-samples *t* tests using the SPSS 18.0 software (SPSS Inc./USA). A value of *P* < 0.05 was considered significant.

## Supplementary Data

Supplementary data are available at *Database* Online.
